# Supplementary material for: Versatile synthesis of metal-compound based mesoporous Janus nanoparticles
Source: Nat Commun. 2023 Jul 17;14:4249. doi: 10.1038/s41467-023-40017-2 (PMC10352278; doi:10.1038/s41467-023-40017-2)
Supplement: Supplementary file 1 — Supplementary Information [file 41467_2023_40017_MOESM1_ESM.pdf]

Supplementary Information

Versatile synthesis of metal-compound based mesoporous Janus nanoparticles

Yan Yu, Runfeng Lin, Hongyue Yu, Minchao Liu, Enyun Xing, Wenxing Wang, Fan Zhang, Dongyuan Zhao and Xiaomin Li*

Department of Chemistry, Shanghai Stomatological Hospital & School of Stomatology, State Key Laboratory of Molecular Engineering of Polymers, iChem, Shanghai Key Laboratory of Molecular Catalysis and Innovative Materials, Fudan University, Shanghai 200433, China.

*Corresponding to Prof. X. Li: lixm@fudan.edu.cn.

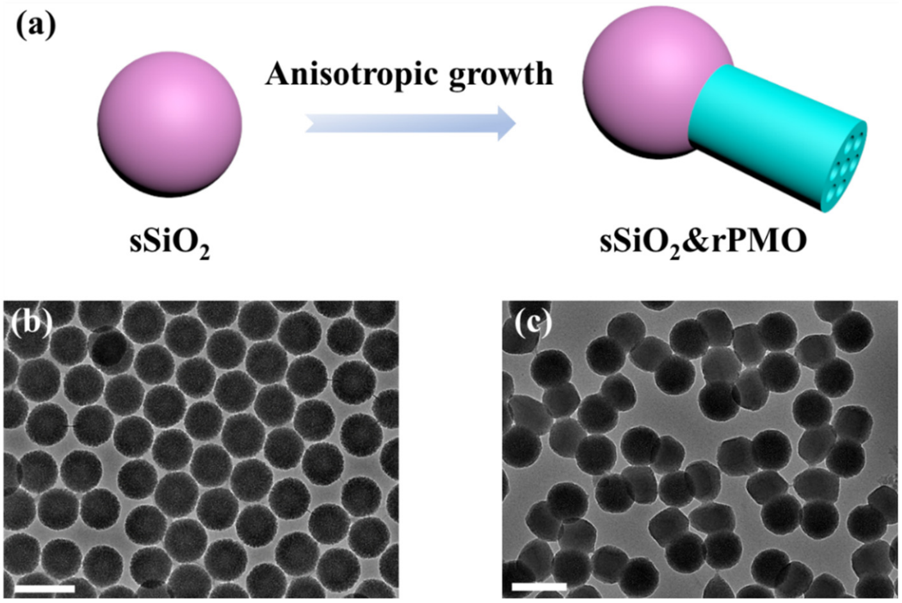

Supplementary Fig. 1 (a) Schematic illustration of the fabrication process of pristine Janus $sSiO_2$&rPMO template. TEM images of (b) $sSiO_2$ and (c) $sSiO_2$&rPMO nanoparticles. The scale bars are 200 nm.

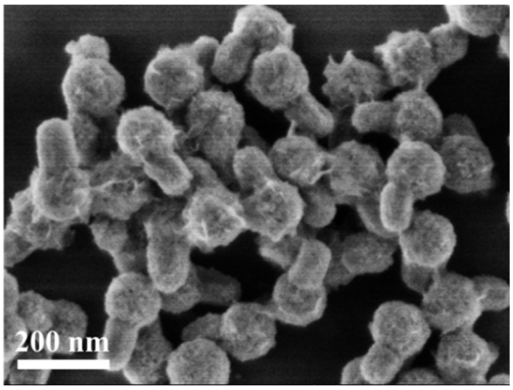

Supplementary Fig. 2 SEM image of Ni-$sSiO_2$&rPMO mJNPs after the selective assembly process.

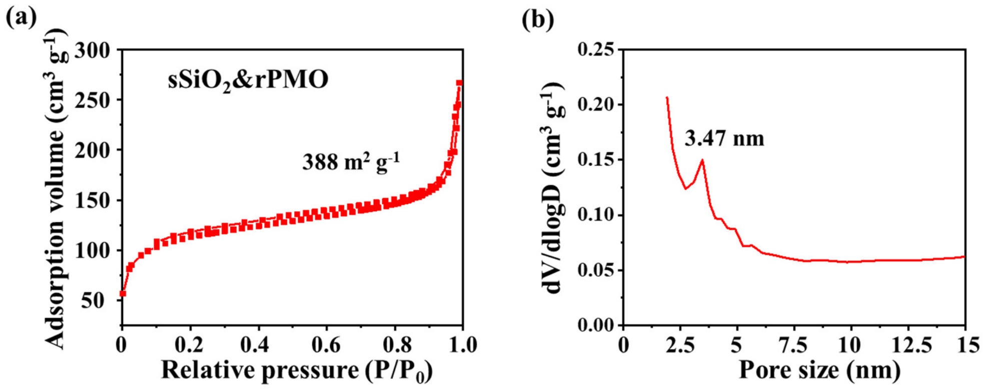

Supplementary Fig. 3 (a) N_2 sorption isotherms and (b) the corresponding pore size distribution curve of pristine $sSiO_2$&rPMO. Source data are provided as a Source Data file.

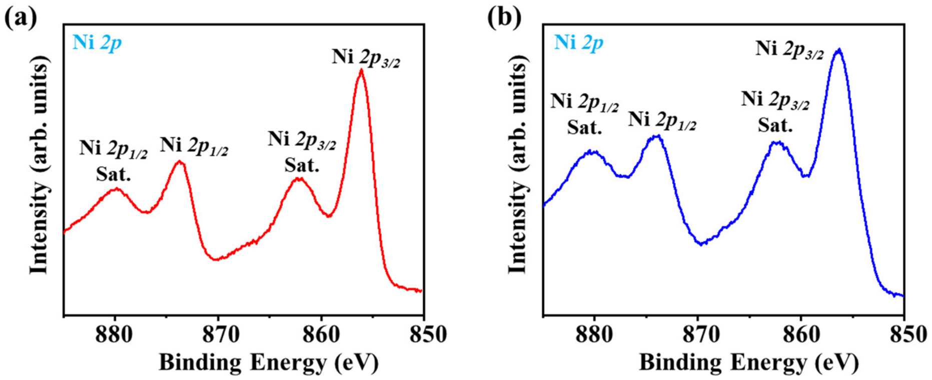

Supplementary Fig. 4 Ni *2p* XPS spectra of the obtained Ni-sSiO_2&rPMO (a) before and (b) after calcination treatment. Source data are provided as a Source Data file.

Either before or after calcination, the Ni-mJNPs show distinctive signals corresponded to Ni^{2+} species. Before calcination, the XPS spectrum of Ni-sSiO_2&rPMO exhibits a sharp peak centered at 856.08 eV corresponding to Ni $2p_{3/2}$ with a satellite at 862.18 eV and a Ni $2p_{1/2}$ peak centered at 873.68 eV with a satellite at 879.88 eV, which is well matched with pure $Ni(OH)_2$ spectrum.[1,2] After calcination, the ratio between the satellite peak and main peak increased, which is in good consistency with the phase transformation of $Ni(OH)_2$ to NiO characterized by XRD. Typical Ni $2p_{3/2}$ peak of pure NiO is asymmetrical with an important contribution at ~ 854 eV. For the Ni-sSiO_2&rPMO after calcination, the Ni $2p_{3/2}$ peak appears symmetrical and shifts to higher binding energy at ~ 856.38 eV, which may be attributed to the strong interaction of Ni^{2+} species with the silica support.[3,4]

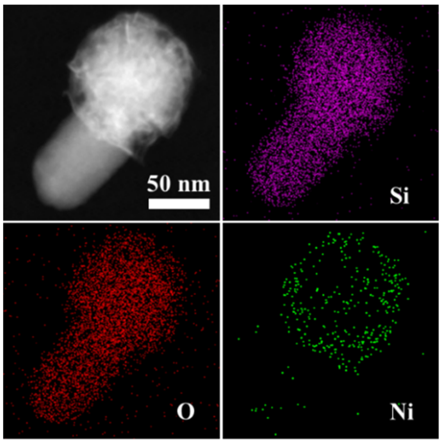

Supplementary Fig. 5 HAADF-STEM and EDS mapping images of Ni-sSiO_2&rPMO mJNPs after calcination.

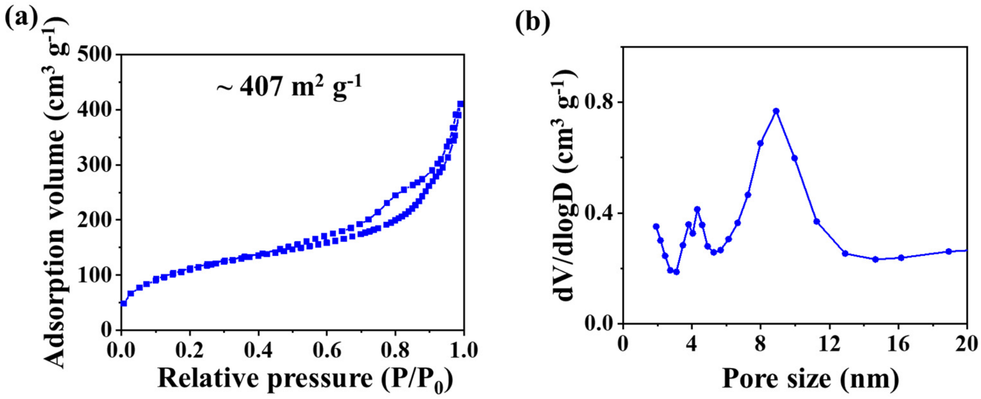

Supplementary Fig. 6 (a) N_2 sorption isotherms and (b) the corresponding pore size distribution curve of the obtained Ni-sSiO_2&rPMO mJNPs after calcination. Source data are provided as a Source Data file.

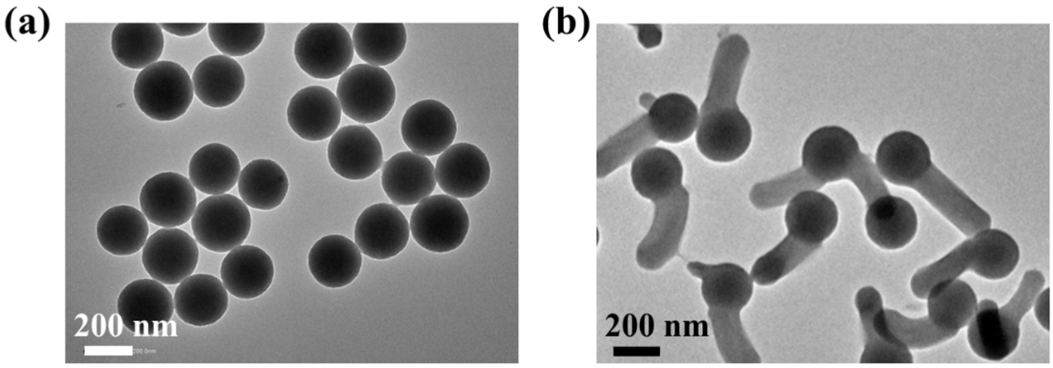

Supplementary Fig. 7 TEM images of (a) hydrophobic sPMO and (b) Janus sPMO&rSiO_2 with spherical hydrophobic PMO head and rod shaped hydrophilic SiO_2 tail.

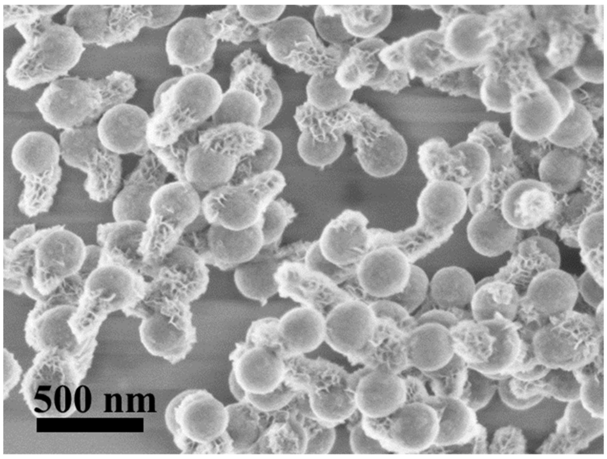

Supplementary Fig. 8 SEM image of sPMO&rSiO_2-Ni mJNPs, in which the $Ni(OH)_2$ nanosheets selectively assemble on the rod-shaped tail of the pristine template.

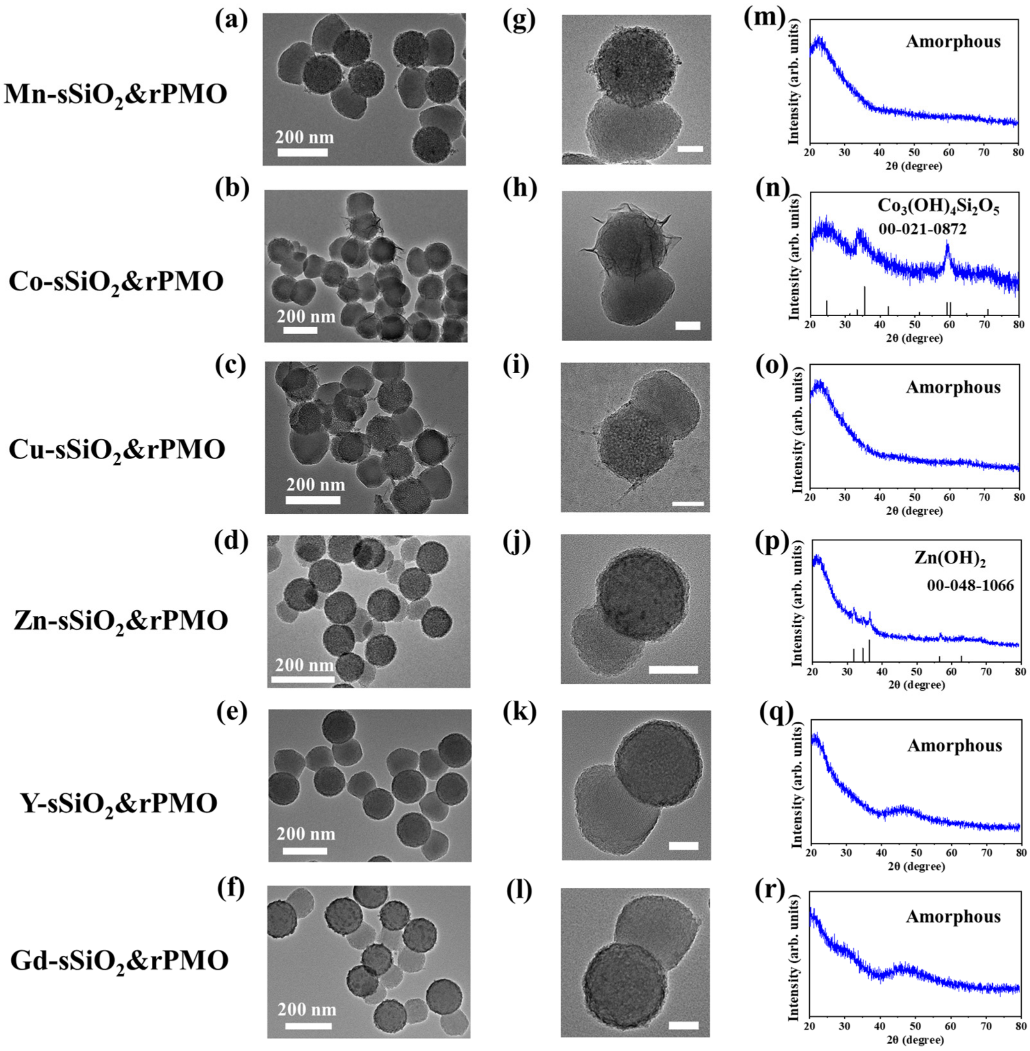

Supplementary Fig. 9 TEM images and XRD patterns of the obtained metal-compound based mJNPs before calcination treatment. (a, g, m) Mn-$sSiO_2$&rPMO; (b, h, n) Co-$sSiO_2$&rPMO; (c, i, o) Cu-$sSiO_2$&rPMO; (d, j, p) Zn-$sSiO_2$&rPMO; (e, k, q) Y-$sSiO_2$&rPMO and (f, l, r) Gd-$sSiO_2$&rPMO. The scale bars in (g-l) are 50 nm. Source data are provided as a Source Data file.

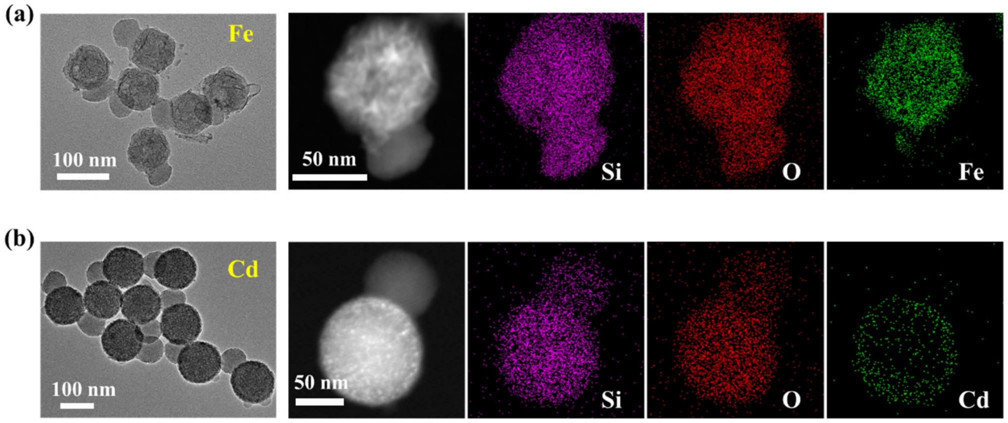

Supplementary Fig. 10 TEM, HAADF-STEM and EDS mapping images of the obtained metal-compound based mJNPs before calcination treatment. (a) Fe-$sSiO_2$&rPMO; (b) Cd-$sSiO_2$&rPMO.

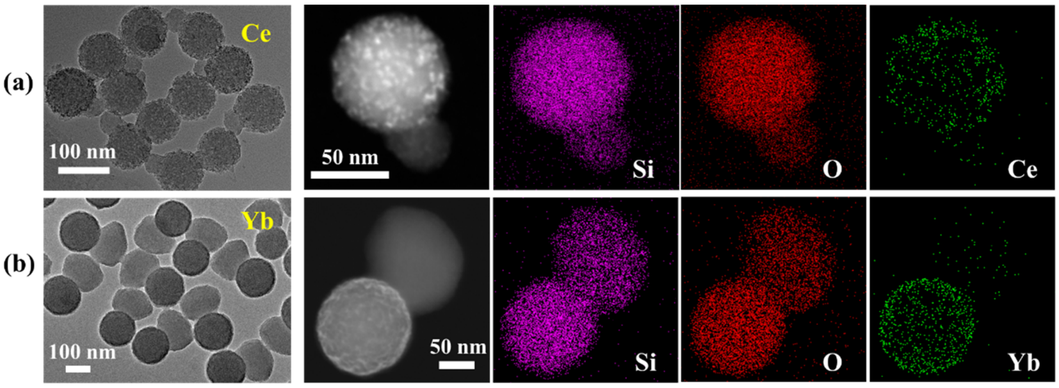

Supplementary Fig. 11 TEM and EDS mapping images of the obtained rare-earth based mJNPs before calcination treatment. (a) Ce-sSiO$_2$&rPMO; (b) Yb-sSiO$_2$&rPMO.

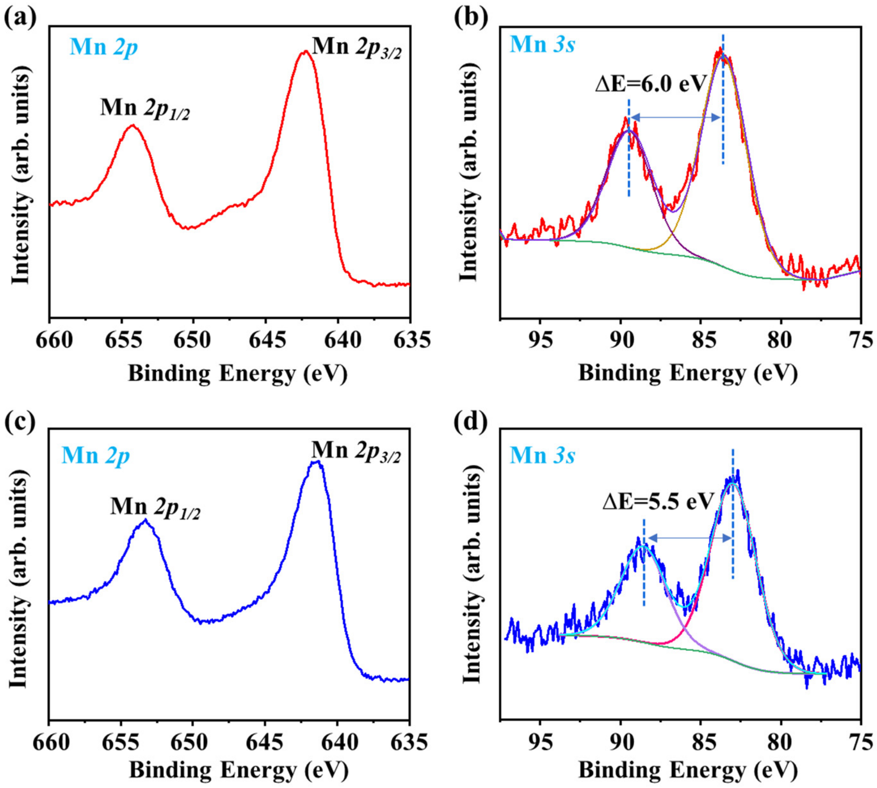

Supplementary Fig. 12 XPS spectra of the obtained Mn-sSiO$_2$&rPMO (a, b) before and (c, d) after calcination treatment. Source data are provided as a Source Data file.

Both the Mn *2p* spectra show significant multiplet splitting feature[5]. The average oxidation state (AOS) of the Mn centers in the Mn-mJNPs was estimated on the basis of the XPS Mn *3s* peak splitting energy (ΔE) using the following correlation[6]: AOS = 8.95-1.13×ΔE (eV).

Before the calcination process, the AOS of Mn in Mn-sSiO$_2$&rPMO is estimated to be 2.28, corresponding to oxidation state of Mn^{2+}. After the calcination, the AOS of Mn in Mn-sSiO$_2$&rPMO was estimated to be 2.74, indicating the transformation of the oxidation state from Mn^{2+} to Mn^{3+}, which is in good consistency with XRD results.

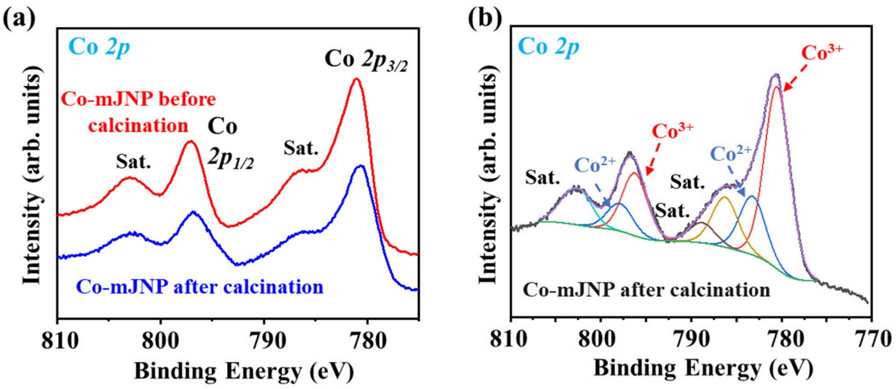

Supplementary Fig. 13 (a) XPS spectra of the obtained Co-sSiO$_2$&rPMO mJNPs before and after calcination treatment. (b) Analysis of XPS spectrum of the Co-sSiO$_2$&rPMO mJNPs after calcination. Source data are provided as a Source Data file.

Both Co *2p* spectra obtained from the samples before and after calcination show significant multiplet splitting peaks and satellite peaks corresponding to oxidation states of Co. For the Co-mJNP after calcination, the Co $2p_{3/2}$ peak can be divided into two peaks at 780.58 and 783.60 eV, which corresponding to Co^{3+} and Co^{2+}, respectively. So as to the Co $2p_{1/2}$ peaks, demonstrating the existence of both Co^{2+} and Co^{3+} oxidation state.[7,8]

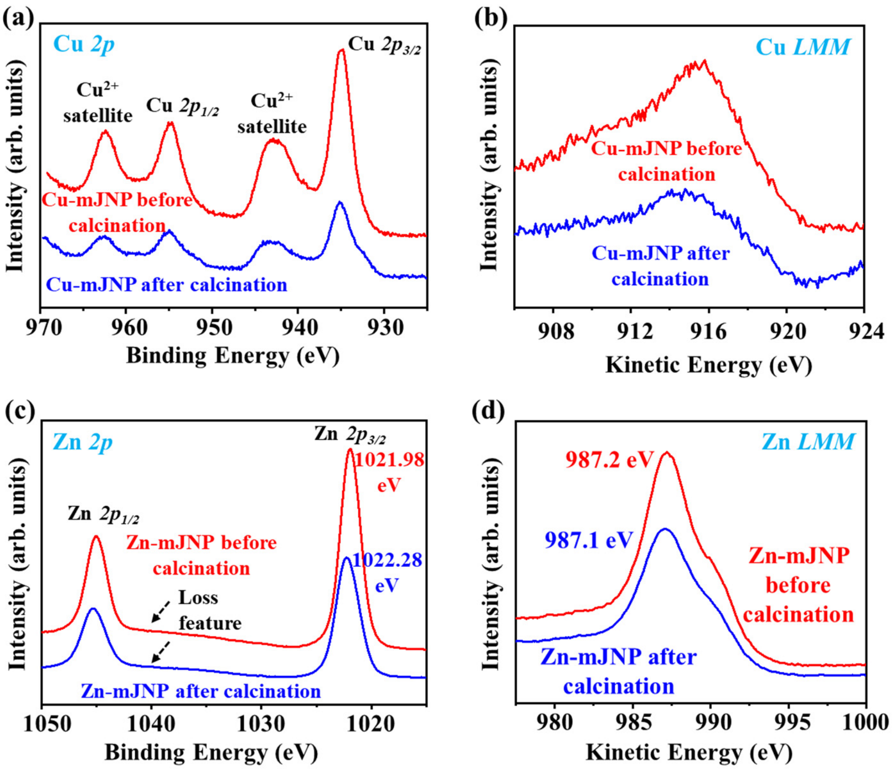

Supplementary Fig. 14 XPS spectra of the obtained Cu-mJNPs and Zn-mJNPs. (a) Cu *2p* and (b) Cu *LMM* Auger spectra of Cu-mJNPs before and after calcination. (c) Zn *2p* and (d) Zn *LMM* Auger spectra of Zn-mJNPs before and after calcination. Source data are provided as a Source Data file.

Both the Cu *2p* spectra of Cu-mJNPs before and after calcination show strong satellite peaks at ~ 943 and ~ 962.68 eV, demonstrating the presence of Cu^{2+} in the metal compound.[9] After the calcination, the multiplet peaks centered at 934.98 and 954.88 eV ascribed to Cu $2p_{3/2}$ and Cu $2p_{1/2}$

slightly shift to 935.18 and 955.08 eV accompanied with shape changes, indicating for the change of the binding environments of Cu^{2+}, which is consistent with the analysis of the kinetic energy.

As for Zn-mJNP, both the Zn *2p* spectra of Zn-mJNPs before and after calcination show distinctive multiplet peaks at ~1022 and ~ 1045 eV ascribed to Zn $2p_{3/2}$ and Zn $2p_{1/2}$, which is consistent with the feature of Zn^{2+}.[10] The Auger parameter (calculated with α'=BE+KE) of Zn-mJNPs increased by 0.2 eV after calcination, which is in accordance with the transformation between the $Zn(OH)_2$ and ZnO indicated by XRD results.

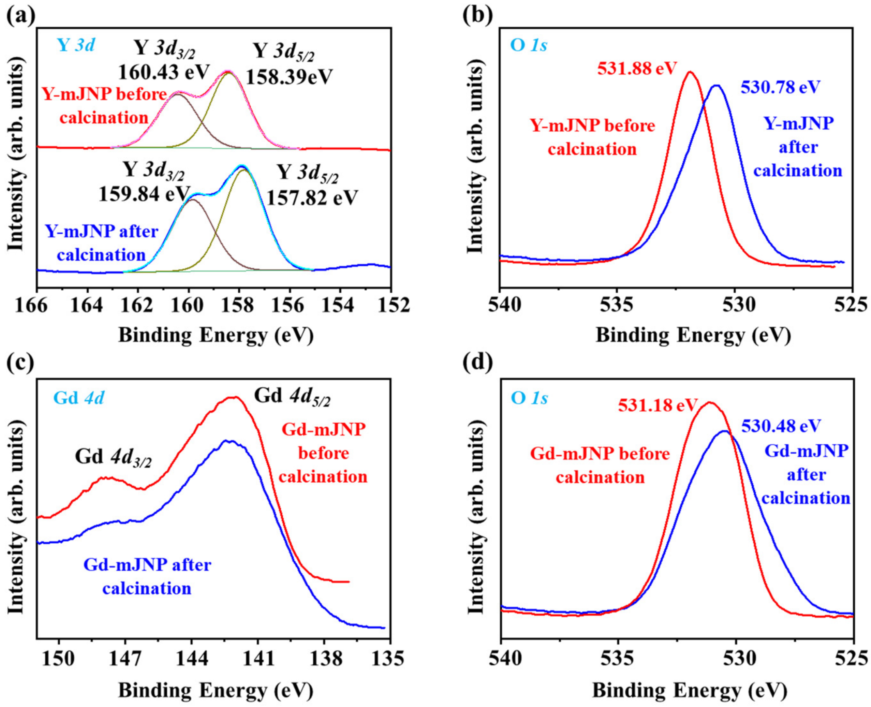

Supplementary Fig. 15 XPS spectra of the obtained Y-mJNPs and Gd-mJNPs. (a) Y *3d* and (b) O *1s* spectra of Y-mJNPs before and after calcination. (c) Gd *3d* and (d) O *1s* spectra of Gd-mJNPs before and after calcination. Source data are provided as a Source Data file.

The Y *3d* spectra suggest the presence of Y^{3+} in the samples before and after calcination. After calcination, the Y $3d_{5/2}$ and Y $3d_{3/2}$ peaks located at 158.39 and 160.43 eV shifted to lower binding energies of 157.82 and 159.84 eV, indicating the transformation of the metal compound from $Y(OH)_3$ to Y_2O_3.[11] In addition, the O *1s* peak shifted from 531.88 to 530.78 eV, further indicating the transformation of hydroxide compound to metal oxide. Combing with the XRD results, the composition of Y-mJNPs before calcination is considered to be amorphous $Y(OH)_3$, which transformed to cubic Y_2O_3 after the calcination.

The Gd *4d* XPS spectra for both samples show peaks at 142.18 and 147.78 eV corresponding to the trivalent Gd^{3+}.[12,13] The O *1s* peak center at 531.18 eV shifted from to 530.48 after calcination, indicating the transformation of hydroxide compound to metal oxide.

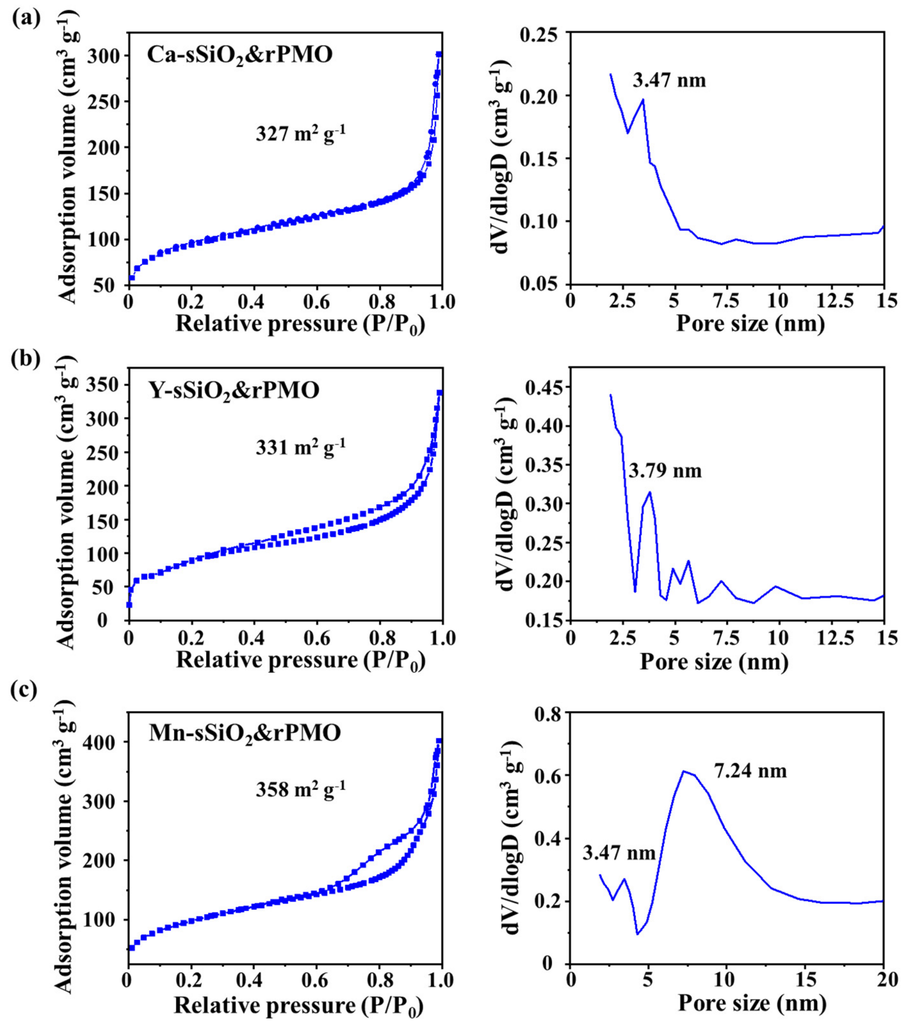

Supplementary Fig. 16 N_2 sorption isotherms and the corresponding pore size distribution curves of the obtained (a) Ca- $sSiO_2$&rPMO, (b) Y-SiO_2&PMO and (c) Mn-$sSiO_2$&PMO after calcination. Source data are provided as a Source Data file.

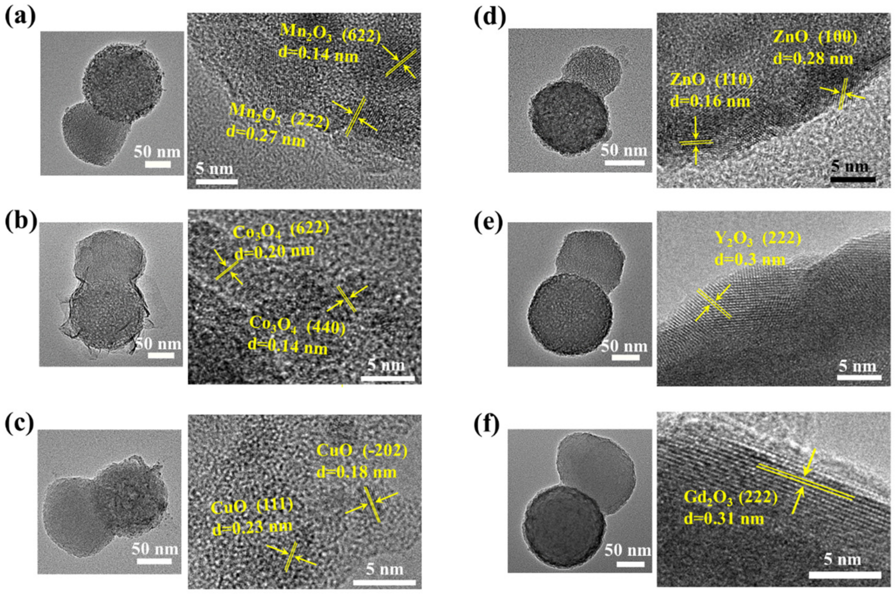

Supplementary Fig. 17 TEM and HRTEM images of the obtained metal-compound based mJNPs after calcination treatment. (a) Mn-sSiO$_2$&rPMO; (b) Co-sSiO$_2$&rPMO; (c) Cu-sSiO$_2$&rPMO; (d) Zn-sSiO$_2$&rPMO; (e) Y-sSiO$_2$&rPMO and (f) Gd-sSiO$_2$&rPMO.

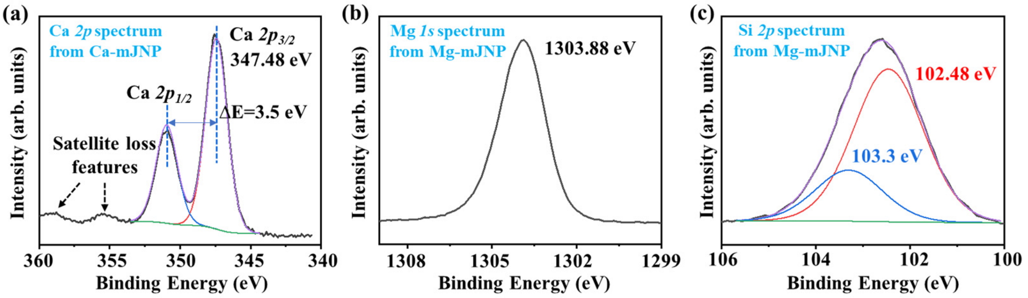

Supplementary Fig. 18 XPS spectra of the obtained Ca-mJNPs and Mg-mJNPs. (a) Ca *2p* spectrum of Ca-mJNPs. (b) Mg *1s* and (c) Si *2p* spectra of Mg-mJNPs. Source data are provided as a Source Data file.

The Ca *2p* spectrum obtained from Ca-sSiO$_2$&rPMO mJNPs shows characteristic peaks corresponding to $CaCO_3$ with multiplet peaks located at 347.48 (Ca $2p_{3/2}$) and 350.98 eV (Ca $2p_{1/2}$) and typical satellite loss features at 355.48 and 358.88 eV. [14]

As for Mg-mJNPs, the binding energy of Mg *1s* in Mg-mJNPs is 1303.88 eV, which is attributed to Mg^{2+} species. The deconvolution of the Si *2p* peak of Mg-sSiO$_2$&rPMO mJNP suggests the presence of two components: one centered at 103.3 eV, attributed to Si *2p* from SiO_2, and another one centered at 102.48 eV, which can be assigned to the Si *2p* from the $MgSiO_3$ moieties.[15] It is in good consistency with the XRD analysis of Mg-sSiO$_2$&rPMO mJNP.

Supplementary Table 1. The metal contents of representative metal-compound based mJNPs determined from ICP analysis.

Element	Ca	Mg	Mn	Co	Ni	Cu	Zn	Y	Gd
Content (wt %)	5.18	5.41	7.50	6.58	5.22	7.11	10.03	5.97	6.10

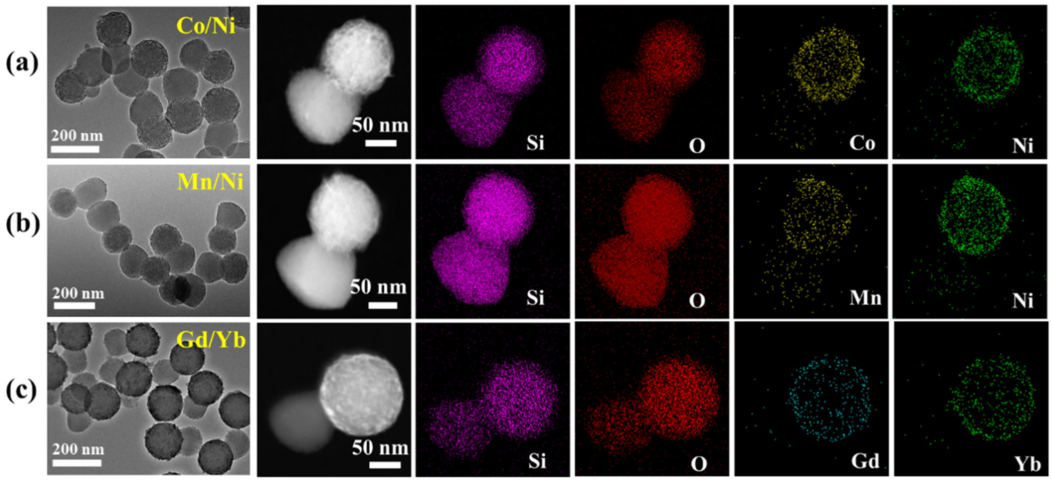

Supplementary Fig. 19 TEM and EDS mapping images of the obtained metal-compound based mJNPs with dual metal elements before calcination treatment. (a) Co/Ni-$sSiO_2$&rPMO; (b) Mn/Ni-$sSiO_2$&rPMO; (c) Gd/Yb-$sSiO_2$&rPMO.

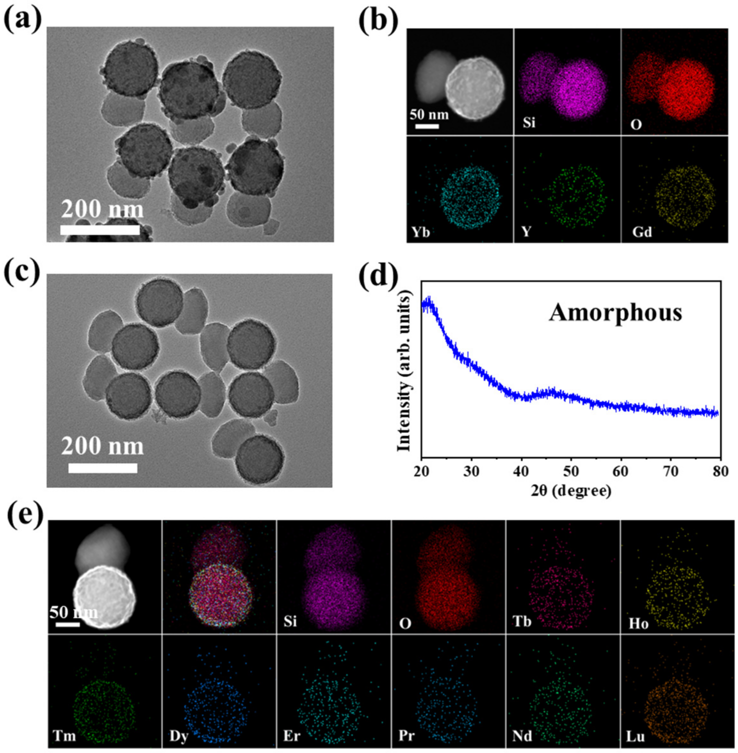

Supplementary Fig. 20 TEM, HAADF-STEM and EDS mapping images, XRD pattern of the obtained metal-compound based mJNPs with multiple metal elements before calcination treatment. (a, b) Yb/Y/Gd- $sSiO_2$&rPMO; (c-e) Tb/Ho/Tm/Dy/Er/Pr/Nd/Lu-$sSiO_2$&rPMO. Source data are provided as a Source Data file.

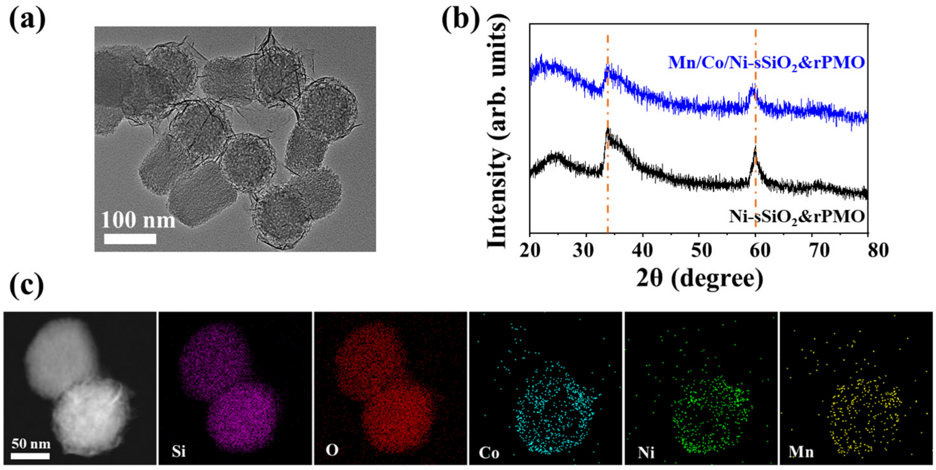

Supplementary Fig. 21 (a) TEM image, (b) XRD pattern and (c) HAADF-STEM and EDS mapping images of ternary Co/Ni/Mn- $sSiO_2$&rPMO mJNPs before calcination treatment. Source data are provided as a Source Data file.

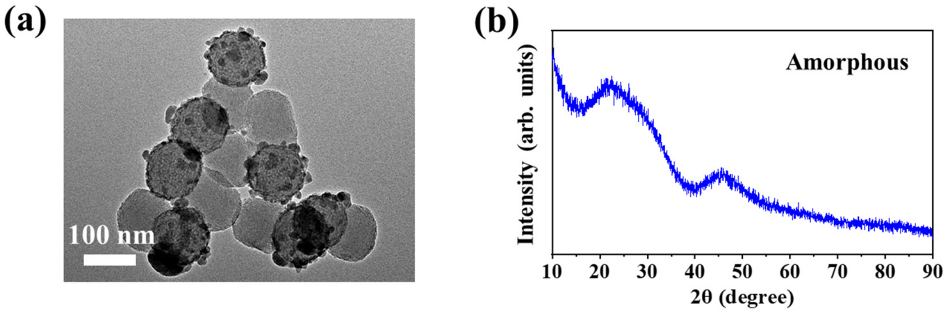

Supplementary Fig. 22 (a) TEM image and (b) XRD pattern of Tm/Y/La/Pr/Tb/Nd/Ho/Dy/Lu-$sSiO_2$&rPMO mJNPs before calcination treatment. Source data are provided as a Source Data file.

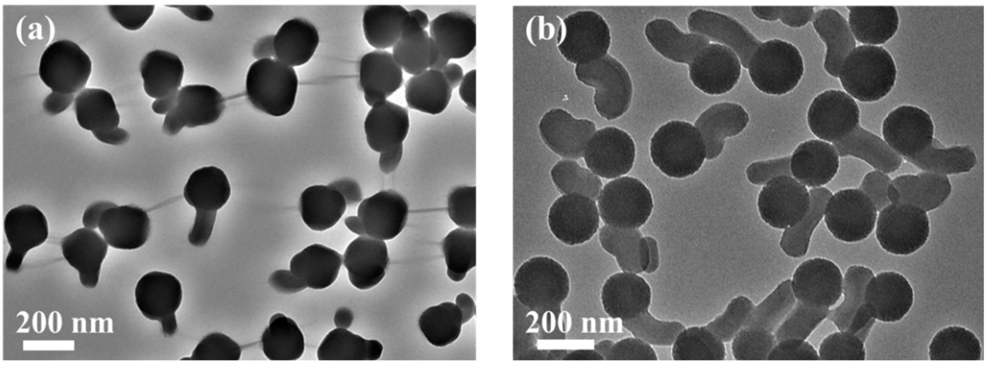

Supplementary Fig. 23 TEM images of the synthesized Janus nanoparticles with isotropic surface properties. (a) hydrophilic $sSiO_2$&$rSiO_2$; (b) hydrophobic sPMO&rPMO.

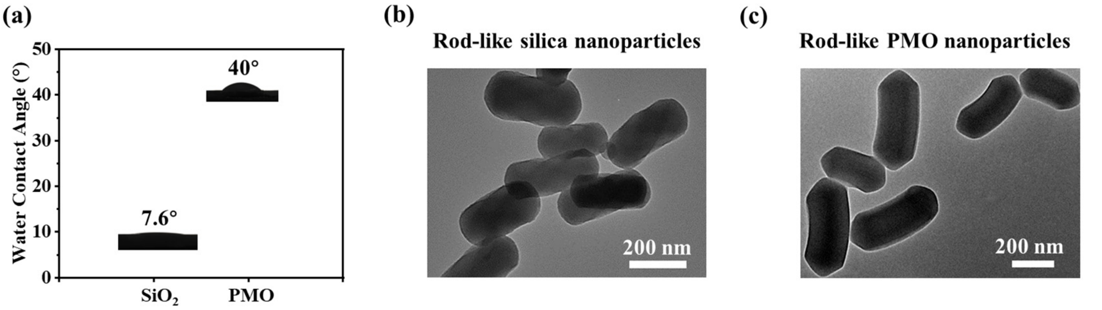

Supplementary Fig. 24 (a) Contact angles of water on the rod-like SiO_2 and PMO. TEM images of (a) rod-like silica nanoparticles and (b) rod-like PMO nanoparticles.

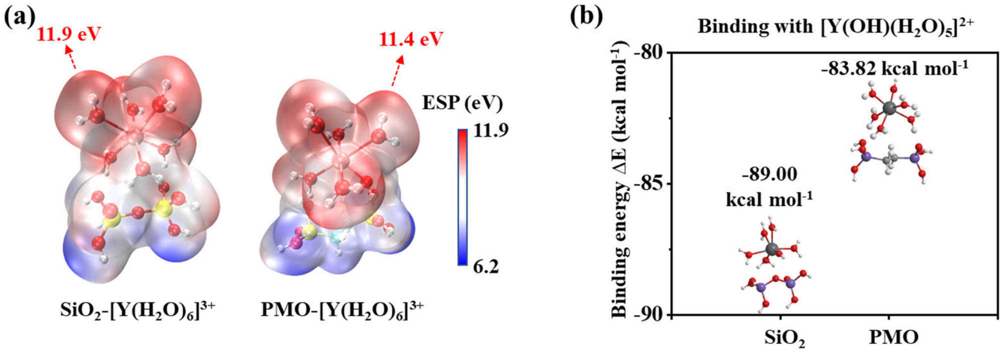

Supplementary Fig. 25 (a) The electrostatic potential mappings of SiO_2-$[Y(H_2O)_6]^{3+}$ and PMO-$[Y(H_2O)_6]^{3+}$ composites. (g) The interaction energies of $[Y(OH)(H_2O)_5]^{2+}$ on SiO_2 and PMO.

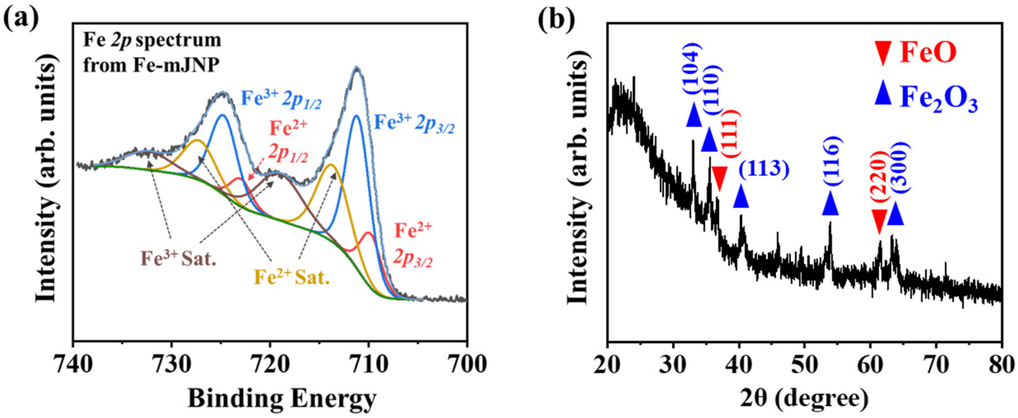

Supplementary Fig. 26 (a) XPS spectrum in the Fe *2p* region and (b) XRD pattern of the obtained Fe-sSiO_2&rPMO mJNPs. Source data are provided as a Source Data file.

According to the XPS spectrum of Fe *2p*, the fitted splitting peaks of Fe $2p_{3/2}$ and Fe $2p_{1/2}$ can be divided into two groups: Fe^{2+} (709.8/722.97 eV) and Fe^{3+} (711.13/724.7 eV). The satellite peaks corresponding to Fe shakeup at the high binding energy side of the Fe $2p_{3/2}$ and Fe $2p_{1/2}$ were ascribed to Fe^{3+} (719.00/732.37 eV) and Fe^{2+} (713.65/727.21 eV).[16] The XRD patterns further demonstrate that the metal compound is composed by FeO and Fe_2O_3. Based on the analysis of the Fe *2p* spectrum via peak fitting, the Fe^{3+}/Fe^{2+} ratio in Fe-mJNPs was calculated to be 1.48.

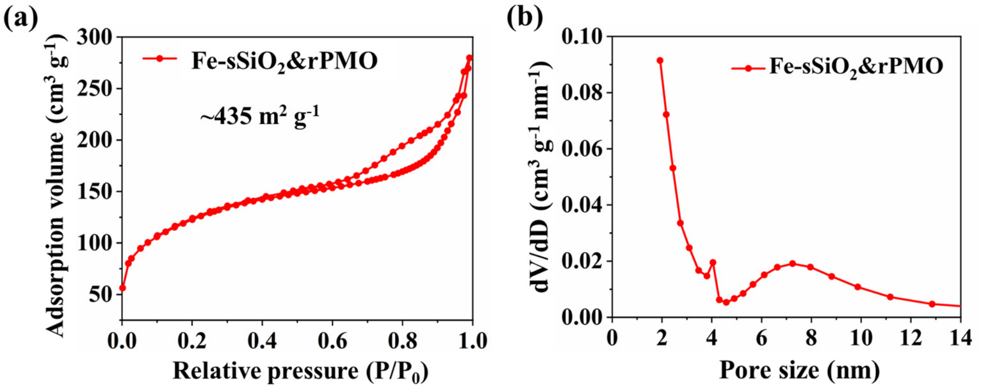

Supplementary Fig. 27 (a) N_2 sorption isotherms and (b) the corresponding pore size distribution curve of the obtained Fe-sSiO$_2$&rPMO mJNPs. Source data are provided as a Source Data file.

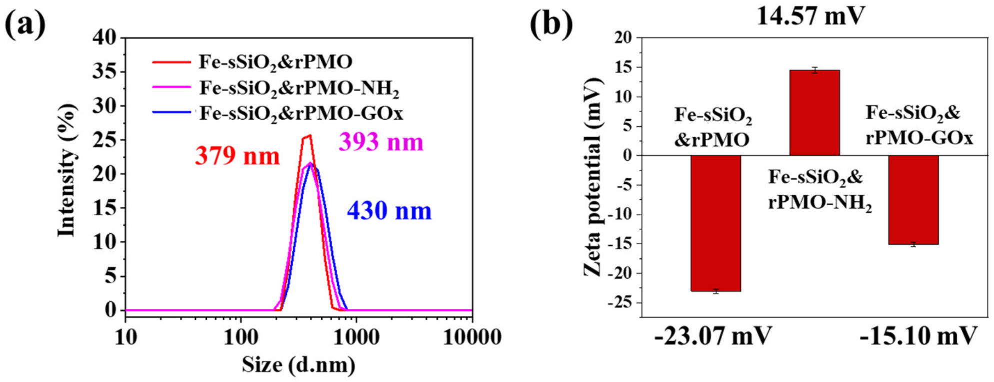

Supplementary Fig. 28 (a) The hydrodynamic diameter and (b) zeta potential of the obtained Fe-sSiO$_2$&rPMO, amino-functionalized Fe-sSiO$_2$&rPMO-NH$_2$ and GOx-grafted Fe-sSiO$_2$&rPMO-GOx mJNPs. Error bars represent means ± SD from three independent experiments. Source data are provided as a Source Data file.

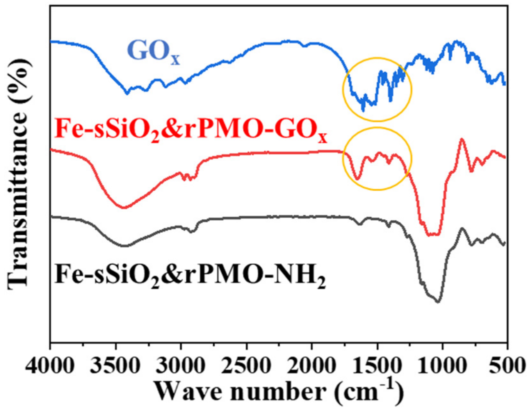

Supplementary Fig. 29 Fourier transform infrared (FTIR) spectra of the GOx, amino-functionalized Fe-sSiO$_2$&rPMO-NH$_2$ and GOx-grafted Fe-sSiO$_2$&rPMO-GOx mJNPs. The yellow circles show the characteristic peaks of GOx, which confirms the successful loading of GOx in Fe-sSiO$_2$&PMO-GOx. Source data are provided as a Source Data file.

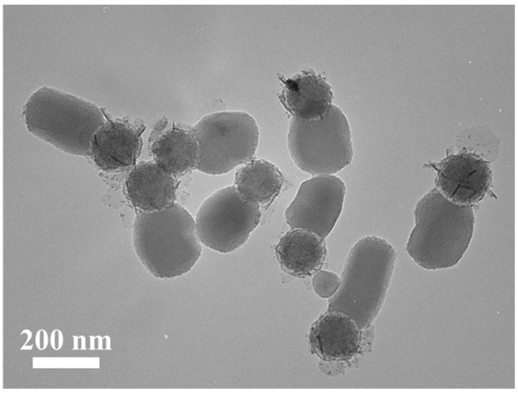

Supplementary Fig. 30 TEM image of the Fe-$sSiO_2$&rPMO-GOx mJNPs.

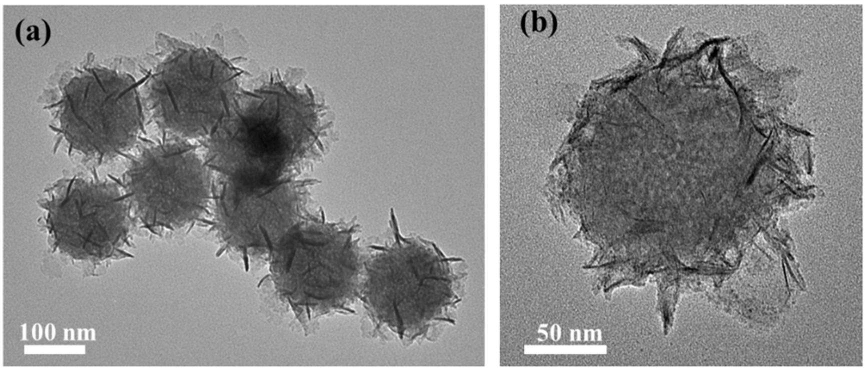

Supplementary Fig. 31 TEM images with different magnifications of the obtained Fe-$sSiO_2$ nanoparticles with the isotropic surface property.

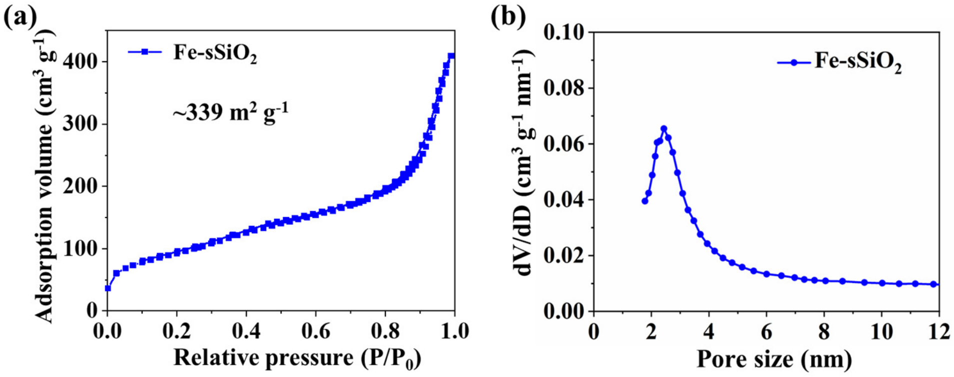

Supplementary Fig. 32 (a) N_2 sorption isotherms and (b) the corresponding pore size distribution of the obtained Fe-$sSiO_2$ nanocomposites. Source data are provided as a Source Data file.

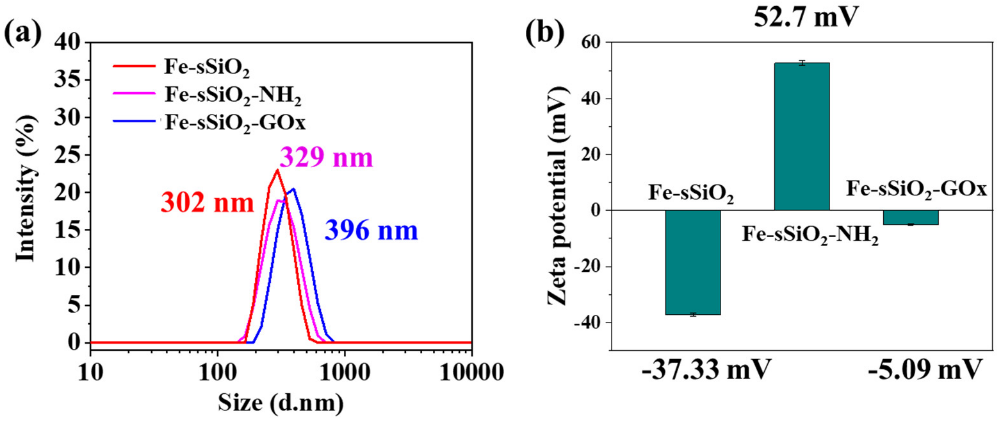

Supplementary Fig. 33 (a) The hydrodynamic diameter and (b) zeta potential of the obtained Fe-$sSiO_2$, amino-functionalized Fe-$sSiO_2$-NH_2 and GOx-grafted Fe-$sSiO_2$-GOx nanocomposites. Error bars represent means ± SD from three independent experiments. Source data are provided as a Source Data file.

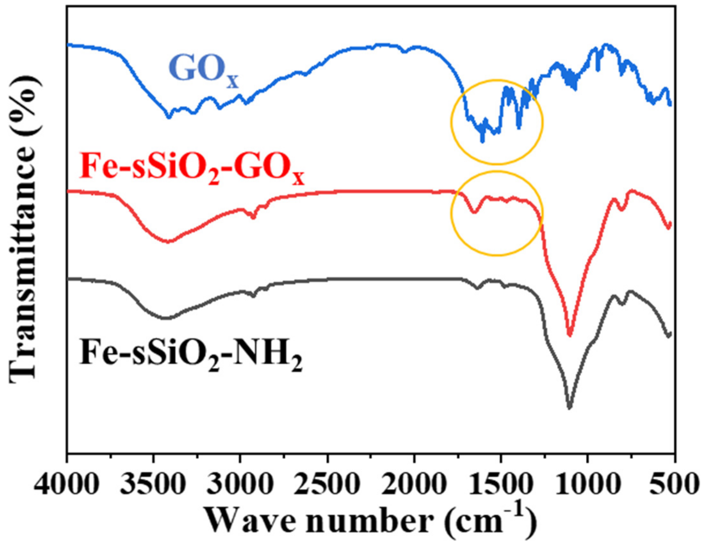

Supplementary Fig. 34 FTIR spectra of the obtained Fe-$sSiO_2$, amino-functionalized Fe-$sSiO_2$-NH_2 and GOx-grafted Fe-$sSiO_2$-GOx nanocomposites. The yellow circles show the characteristic peaks of GOx, which confirms the successful loading of GOx in Fe-$sSiO_2$-GOx.Source data are provided as a Source Data file.

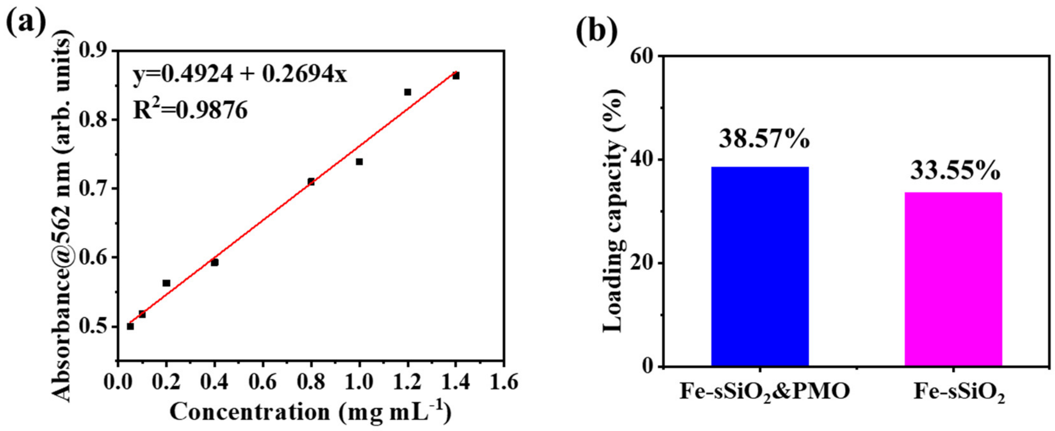

Supplementary Fig. 35 (a) The standard curve of absorption intensity at 562 nm of GOx as a function of concentration. (b) The loading capacity of GOx in the nanocomposites, which were determined by the ratio between the grafting amount of GOx and the weight of Fe element in the nanocomposites. Source data are provided as a Source Data file.

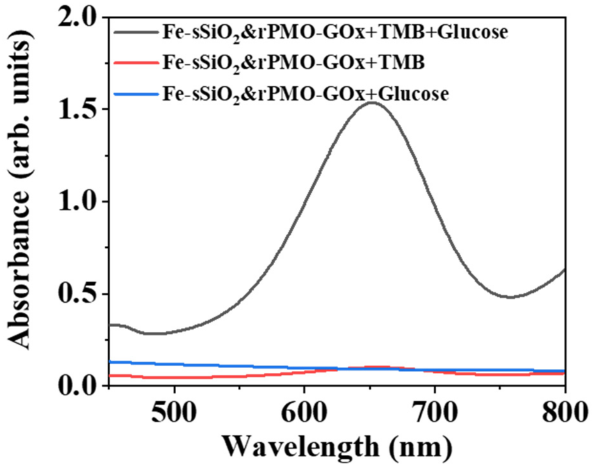

Supplementary Fig. 36 UV-vis absorption spectra of the oxTMB products of the catalytic reaction under different conditions. Source data are provided as a Source Data file.

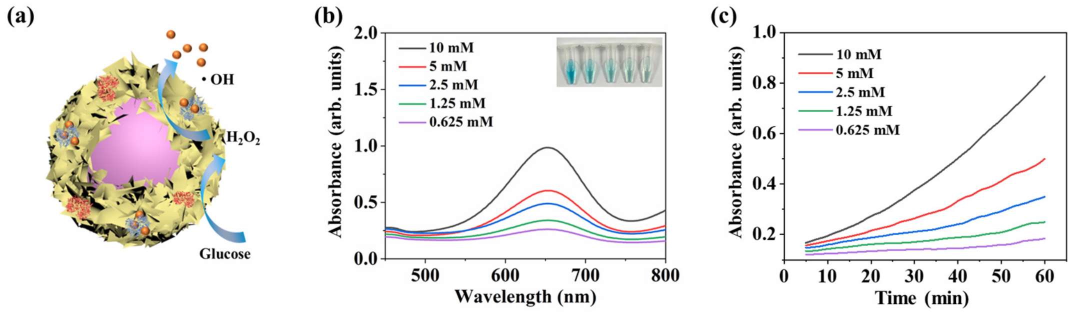

Supplementary Fig. 37 (a) Schematic illustration of cascade nanocatalysts based on Fe-sSiO$_2$-GOx nanocomposites. In this case, the as produced •OH can easily contact with GOx enzyme, thus suffering from adverse effect of strong oxidizing •OH on the activity and stability of GOx. (b) UV-

vis absorption spectra of the TMB solutions under catalysis of Fe-sSiO$_2$-GOx (10 μg/mL) upon the addition of varied concentrations of β-D-glucose (10, 5, 2.5, 1.25, and 0.625 mM) for 1h. (c) Time-dependent absorbance changes at 650 nm of TMB solutions under the catalysis of Fe-sSiO$_2$-GOx nanocomposites with varied β-D-glucose concentrations (10, 5, 2.5, 1.25 and 0.625 mM). Source data are provided as a Source Data file.

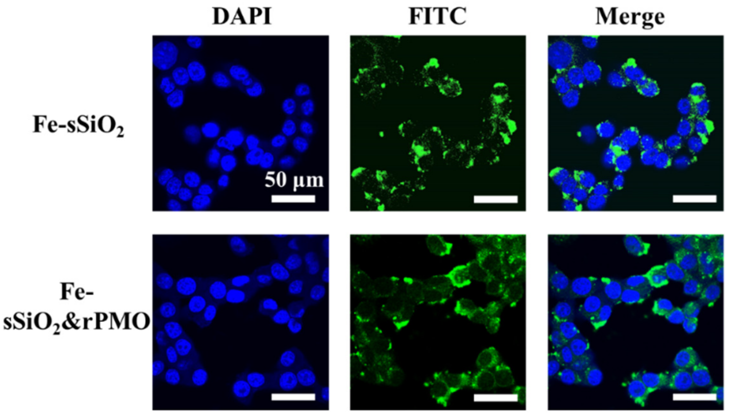

Supplementary Fig. 38 Intracellular uptake of nanocomposites with difference architectures. Representative confocal laser scanning microscopy (CLSM) images of HepG2 cells after co-incubation with FITC-labeled nanocomposites at 37 °C for 6 h. Scale bars are 50 μm.

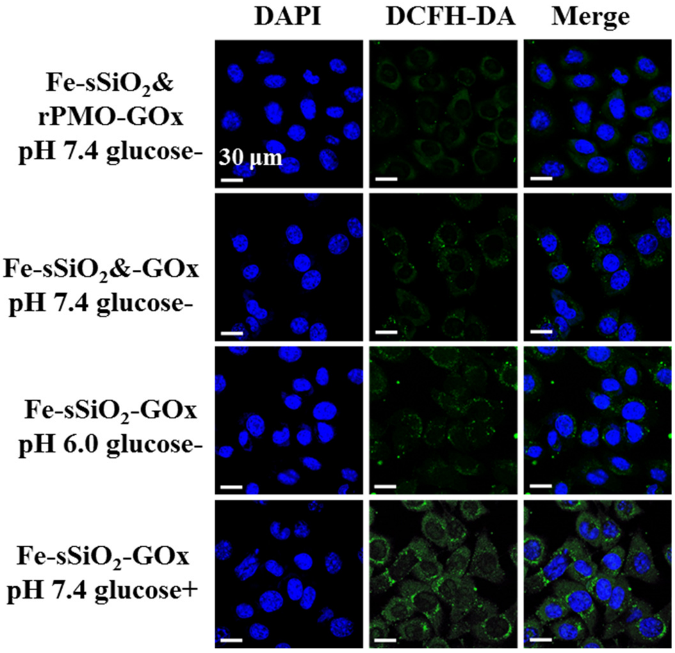

Supplementary Fig. 39 CLSM images of ROS fluorescence probe in the HepG2 cell. The cells were co-incubated with Fe-sSiO$_2$&PMO-GOx or Fe-sSiO$_2$-GOx in the presence and absence of glucose (5 mM) at pH 7.4 and 6.0 for 4 h. Scale bars are 30 μm.

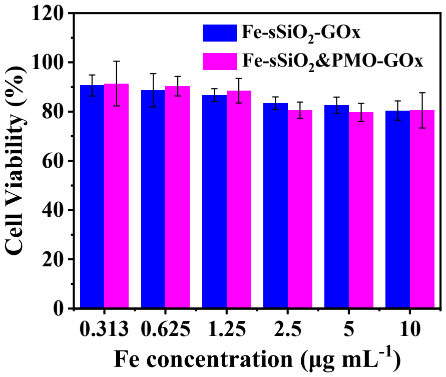

Supplementary Fig. 40 Cell viability of HUVEC cells after co-incubation with the nanocomposites with difference architectures. The results show that both the Fe-sSiO$_2$&PMO-GOx and Fe-sSiO$_2$-GOx nanocatalysts exhibit negligible cytotoxicity to normal cells. Error bars represent means ± SD from three independent experiments. Source data are provided as a Source Data file.

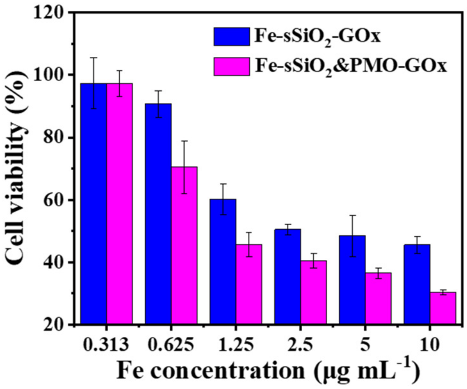

Supplementary Fig. 41 Cell viability of HepG2 cells after co-incubation with the nanocatalysts with difference architectures. The cell viabilities decrease as the increase of nanocatalysts' concentration and the cancer cell killing efficiency of the Fe-sSiO$_2$&PMO-GOx nanocatalysts is higher than that of the Fe-sSiO$_2$-GOx nanocatalysts at each concentration. Error bars represent means ± SD from three independent experiments. Source data are provided as a Source Data file.

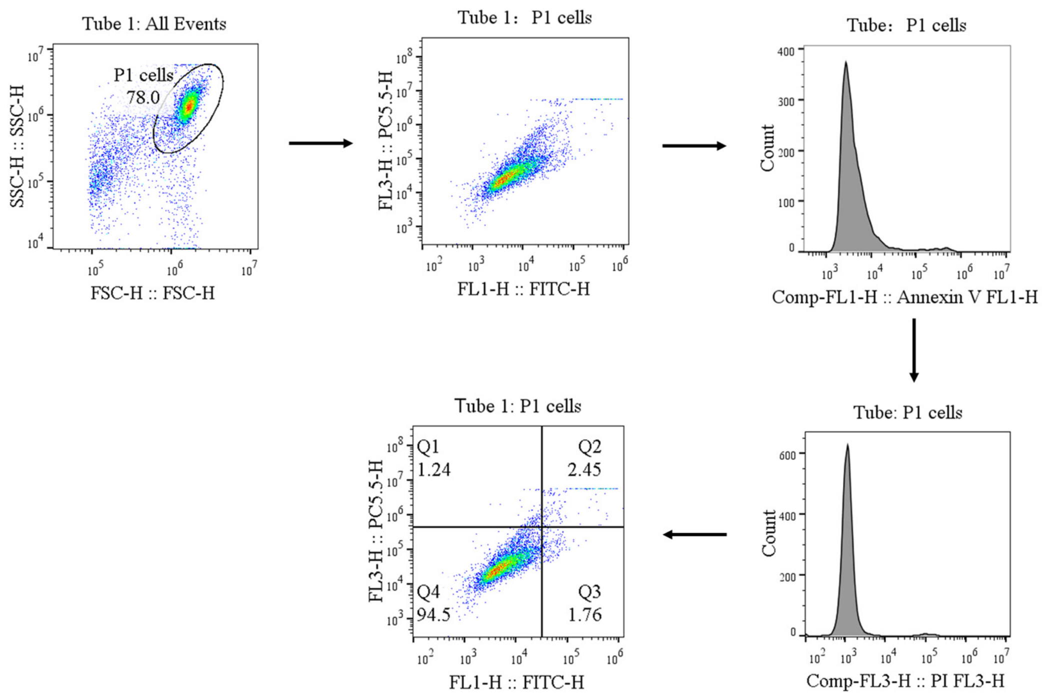

Supplementary Fig. 42 Representative image showing gating strategy for flow cytometry in HepG2 cell.

Supplementary References

1. Biesinger, M. C. et al. X-ray photoelectron spectroscopic chemical state quantification of mixed nickel metal, oxide and hydroxide systems. *Surf. Interface Anal.* **2009,** *41,* 324-332.

2. Ang, M. L. et al. Highly Active Ni/xNa/CeO_2 Catalyst for the Water-Gas Shift Reaction: Effect of Sodium on Methane Suppression. *ACS Catal.* **2014,** *4,* 3237-3248.

3. El-Safty, S. A. et al. Nanosized NiO particles wrapped into uniformly mesocaged silica frameworks as effective catalysts of organic amines. *Appl. Catal. A-Gen.* **2008,** *337,* 121-129.

4. Brussino, P. et al. Tuning the properties of NiO supported on silicon-aluminum oxides: Influence of the silica amount in the ODH of ethane. *Catal. Today* **2022,** *394-396,* 133-142.

5. Biesinger, M. C. et al. Resolving surface chemical states in XPS analysis of first row transition metals, oxides and hydroxides: Cr, Mn, Fe, Co and Ni. *Appl. Surf. Sci.* **2011,** *257*, 2717-2730.

6. Mateos, M. et al. Accessing the Two-Electron Charge Storage Capacity of MnO_2 in Mild Aqueous Electrolytes. *Adv. Energy Mater.* **2020,** *10,* 2000332.

7. Bai, X. et al. Hierarchical Co_3O_4@$Ni(OH)_2$ core-shell nanosheet arrays for isolated all-solid state supercapacitor electrodes with superior electrochemical performance. *Chem. Eng. J.* **2017,** *315,* 35-45.

8. Ning, F. et al. Co_3O_4@layered double hydroxide core/shell hierarchical nanowire arrays for enhanced supercapacitance performance. *Nano Energy* **2014,** *7,* 134-142.

9. Biesinger, M. C. et al. Resolving surface chemical states in XPS analysis of first row transition metals, oxides and hydroxides: Sc, Ti, V, Cu and Zn. *Appl. Surf. Sci.* **2010,** *257*, 887-898.

10. Duchoslav, J. et al. XPS study of zinc hydroxide as a potential corrosion product of zinc: Rapid X-ray induced conversion into zinc oxide. *Corros. Sci.* **2014,** *82,* 356-361.

11. Reddy, I. N. et al. Structural, optical, and XPS studies of doped yttria for superior water splitting under visible light illumination. *J. Electroanal. Chem.* **2019,** *848,* 113335.

12. Nosrati, H. et al. Enhanced In Vivo Radiotherapy of Breast Cancer Using Gadolinium Oxide and Gold Hybrid Nanoparticles. *ACS Appl. Bio Mater.* **2023,** *6,* 784-792.
13. Yin, J. et al. Silica Nanoparticles Decorated with Gadolinium Oxide Nanoparticles for Magnetic Resonance and Optical Imaging of Tumors. *ACS Appl. Nano Mater.* **2021,** *4,* 3767-3779.
14. Moulder, J. F. et al. Handbook of X-ray Photoelectron Spectroscopy, Perkin-Elmer Corp., Eden Prairie, MN, 1992.
15. Brambilla, R. et al. An investigation on structure and texture of silica-magnesia xerogels. *J. Sol-Gel Sci. Techn.* **2009,** *51,* 70-77.
16. Du, W. et al. Fe_3O_4 Mesocrystals with Distinctive Magnetothermal and Nanoenzyme Activity Enabling Self-Reinforcing Synergistic Cancer Therapy. *ACS Appl. Mater. Interfaces* **2020,** *12,* 19285-19294.
