# Supplementary material for: Versatile synthesis of metal-compound based mesoporous Janus nanoparticles
Source: Nat Commun. 2023 Jul 17;14:4249. doi: 10.1038/s41467-023-40017-2 (PMC10352278; doi:10.1038/s41467-023-40017-2)
Supplement: Supplementary file 3 — Description of Additional Supplementary Files [file 41467_2023_40017_MOESM3_ESM.pdf]

Description of Additional Supplementary Files

File Name: Supplementary Data 1
Description: XYZ coordinates of the optimized computational model for water molecule on SiO_2 and PMO calculated via DFT simulation (Fig. 4e).

File Name: Supplementary Data 2
Description: XYZ coordinates of the optimized computational model for $[Ni(OH)(H_2O)_3]^+$ on SiO_2 and PMO calculated via DFT simulation (Fig. 4g).

File Name: Supplementary Data 3
Description: XYZ coordinates of the optimized computational model for $[Y(OH)(H_2O)_5]^{2+}$ on SiO_2 and PMO calculated via DFT simulation (Supplementary Fig. 25b).
